# Diaporthalean fungi associated with canker and dieback of trees from Mount Dongling in Beijing, China

**DOI:** 10.3897/mycokeys.59.38055

**Published:** 2019-10-16

**Authors:** Haiyan Zhu, Meng Pan, Guido Bonthond, Chengming Tian, Xinlei Fan

**Affiliations:** 1 The Key Laboratory for Silviculture and Conservation of Ministry of Education, Beijing Forestry University, Beijing 100083, China Beijing Forestry University Beijing China; 2 GEOMAR Helmholtz Centre for Ocean Research Kiel, Düsternbrooker Weg 20, 24105, Kiel, Germany GEOMAR Helmholtz Centre for Ocean Research Kiel Kiel Germany

**Keywords:** Ascomycota, Diaporthales, new species, phylogeny, taxonomy

## Abstract

Diaporthales is a fungal order comprising important plant pathogens, saprobes and endophytes on a wide range of woody hosts. It is often difficult to differentiate the pathogens in this order, since both the morphology and disease symptoms are similar among the various species. In the current study, we obtained 15 representative diaporthalean isolates from six tree hosts belonging to plant families Betulaceae, Fagaceae, Juglandaceae, Rosaceae, and Ulmaceae from Mount Dongling in China. Six species were identified residing in four families of Diaporthales (Diaporthaceae, Erythrogloeaceae, Juglanconidaceae and Melanconidaceae). Based on morphological comparison and the phylogenetic analyses of partial ITS, LSU, *cal*, *his3*, *rpb2*, *tef1-α* and *tub2* gene sequences, we identified five known species (*Diaporthe betulina*, *D. eres*, *D. rostrata*, *Juglamconis oblonga* and *Melanconis stilbostoma*) and one novel species (*Dendrostoma donglinensis*). These results represent the first study of diaporthalean fungi associated with canker and dieback symptoms from Mount Dongling in Beijing, China.

## Introduction

Diaporthales is an important order in class Sordariomycetes containing taxa that have broad host ranges and widely distributed as plant pathogens, endophytes or saprobes (Fan et al. 2018a, Crous et al. 2019). Most families of the Diaporthales are responsible for diseases on a wide range of host plants, some of which are economically important worldwide, causing anthracnose, blights, cankers, dieback, leaf spots and rots of root and fruit (Alvarez et al. 2016, Guarnaccia and Crous 2017, Voglmayr et al. 2017, Jiang et al. 2019a, Xavier et al. 2019, Fan et al. 2020). The order is characterized by perithecia often with elongate beaks, immersed in stromatic tissues, producing deliquescent paraphyses and unitunicate asci that generally deliquesce, become detached from the perithecial wall when mature, and have a characteristic refractive apical annulus in sexual morph; and acervuli, pycnidia or rarely synnemata, producing phialidic or annellidic conidiogenous cells with 0–1-septate conidia in asexual morph (Barr 1978, Rossman et al. 2007, Fan et al. 2020). The classification of Diaporthales has been confused over the past decades because of the wide variation in morphological characters. Several recent studies have helped to resolve taxonomic problems of Diaporthales by multigene phylogenetic analyses and accepted 30 families in the order (Senanayake et al. 2017, 2018, Braun et al. 2018, Fan et al. 2018a, Crous et al. 2019, Guterres et al. 2019, Xavier et al. 2019).

Mount Dongling has a high diversity of plant species in western Beijing, which is considered as a biodiversity hotspot with more than 1000 plant species (Ma et al. 1995). As more plant species were recorded in this region, the exploration of fungal diversity gradually increased as most fungi are often linked to particular host plants as parasites or endophytes. *Alternaria*, *Diaporthe*, *Leptostroma*, *Pestalotiopsis* and *Phoma* were the most commonly isolated endophytic fungi from *Pinus tabuliformis*, and later additional 38 endophytic taxa were identified from *Acer truncatum* from the Mount Dongling (Guo et al. 2008, Sun et al. 2011). Further, pathogens of Botryosphaeriales have been identified from Mount Dongling, including species from the genera *Aplosporella*, *Botryosphaeria* and *Phaeobotryon* (Zhu et al. 2018).

During the trips to collect forest pathogens causing canker or dieback symptoms in Mount Dongling in Beijing, several specimens associated with typical diaporthalean symptoms were collected from various tree hosts, i.e. *Betula dahurica* (Betulaceae), *Juglans regia*, *J. mandshurica* (Juglandaceae), *Prunus davidiana* (Rosaceae) and *Quercus mongolica* (Fagaceae). As the higher-level phylogeny of many genera within the diaporthalean taxa remains largely unresolved in this region, the current study aims to clarify the systematics and taxonomy of these diaporthalean fungi with detailed descriptions.

## Materials and methods

### Sampling and isolation

**Figure 1.**
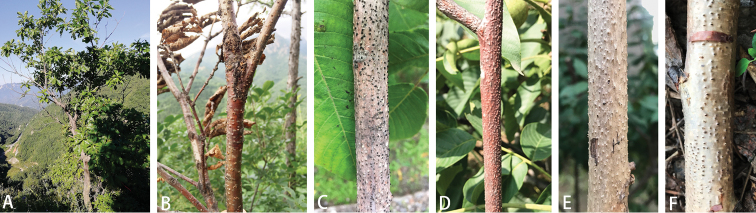
Disease symptoms associated with diaporthalean species. **A, B***Quercus mongolica***C***Juglans regia***D, E***Juglans mandshurica***F***Betula dahurica*.

Fresh specimens of diaporthalean fungi were collected from infected branches of six hosts from Mount Dongling in Beijing, China (Table 1), during the course of cognitive practice at the Beijing Forestry University (**BJFU**). Diaporthalean canker symptoms include elongated, slightly sunken and discolored areas in the bark, which often splits along the canker margin, forming several prominent dark sporocarps immersed and erumpent through the surface of the bark (Fig. 1). A total of 15 isolates were obtained by removing the mucoid spore mass from conidiomata or ascomata of fresh material, which was cut horizontally with a sterile blade and mixed in a drop of sterile water on a glass slide. The contents were broken up further with the blade until a spore suspension was obtained. The suspension was spread over the surface of 1.8 % potato dextrose agar (PDA). Single germinating spores were transferred on to fresh PDA plates. Specimens and isolates were deposited in the Key Laboratory for Silviculture and Conservation of the Ministry of Education in BJFU, and the working Collection of X.L. Fan (**CF**) housed at the BJFU. Axenic cultures are maintained in the China Forestry Culture Collection Centre (**CFCC**).

**Table 1.** Isolates and GenBank accession numbers obtained from Mount Dongling in the current study. (NA – not applicable).

Species	Strain	Host	GenBank accession numbers						
			ITS	LSU	*Cal*	*his3*	*rpb2*	*tef1-α*	*tub2*
*Dendrostoma donglinensis*	CFCC 53148	*Quercus mongolica*	MN266206	MN265880	NA	NA	MN315491	MN315480	NA
	CFCC 53149	*Quercus mongolica*	MN266207	MN265881	NA	NA	MN315492	MN315481	NA
	CFCC 53150	*Quercus mongolica*	MN266208	MN265882	NA	NA	MN315493	MN315482	NA
*Diaporthe betulina*	CFCC 53144	*Betula dahurica*	MN266200	MN265874	MN315462	MN315465	MN315498	MN315474	MN315470
*Diaporthe eres*	CFCC 53145	*Prunus davidiana*	MN266202	MN265876	NA	NA	MN315500	MN315476	MN315472
	CFCC 53146	*Prunus davidiana*	MN266201	MN265875	NA	MN315466	MN315499	MN315475	MN315471
	CFCC 53147	*Juglans regia*	MN266203	MN265877	NA	MN315467	MN315501	MN315477	MN315473
*Diaporthe rostrata*	CFCC 53142	*Juglans mandshurica*	MN266204	MN265878	MN315463	NA	MN315489	MN315478	MN315468
	CFCC 53143	*Juglans mandshurica*	MN266205	MN265879	MN315464	NA	MN315490	MN315479	MN315469
*Juglanconis oblonga*	CFCC 53151	*Juglans mandshurica*	MN266209	MN265883	NA	NA	MN315502	MN315483	NA
	CFCC 53152	*Juglans mandshurica*	MN266210	MN265884	NA	NA	MN315503	MN315484	NA
*Melanconis stilbostoma*	CFCC 53128	*Betula dahurica*	MN266211	MN265885	NA	NA	MN315494	MN315485	NA
	CFCC 53129	*Betula dahurica*	MN266212	MN265886	NA	NA	MN315495	MN315486	NA
	CFCC 53130	*Betula* sp.	MN266213	MN265887	NA	NA	MN315496	MN315487	NA
	CFCC 53131	*Betula* sp.	MN266214	MN265888	NA	NA	MN315497	MN315488	NA

### Morphology

Descriptions were performed based on morphological features of the ascomata or conidiomata from infected host materials. The macro-morphological photographs were captured using a Leica stereomicroscope (M205 FA) (structure and size of stromata, structure and size of ectostromatic disc and ostioles). Micro-morphological observations (shape and size of conidiophores, asci and conidia/ascospores) were determined under a Nikon Eclipse 80i microscope equipped with a Nikon digital sight DS-Ri2 high definition colour camera, using differential interference contrast (DIC) illumination and the Nikon software NIS-Elements D Package v. 3.00. Adobe Bridge CS v. 6 and Adobe Photoshop CS v. 5 were used for the manual editing. Over 10 conidiomata/ascomata, 10 asci and 30 conidia/ascospores were measured to calculate the mean size/length and respective standard deviations (SD). Colony diameters were measured and the colony features were described using the color charts of Rayner (1970). Nomenclatural novelties and descriptions were deposited in MycoBank (Crous et al. 2004).

### DNA isolation, amplification and sequencing

Genomic DNA was extracted from colonies grown on cellophane-covered PDA using a modified CTAB method (Doyle and Doyle 1990). The primers and PCR conditions are listed in Table 2. DNA sequencing was performed using an ABI PRISM 3730XL DNA Analyser with a BigDye Terminator Kit v.3.1 (Invitrogen, USA) at the Shanghai Invitrogen Biological Technology Company Limited (Beijing, China). The DNA sequences obtained from forward and reverse primers were combined using SeqMan v. 7.1.0 in the DNASTAR Lasergene Core Suite software (DNASTAR Inc., Madison, WI, USA). Reference sequences were selected based on ex-type or ex-epitype sequences available from relevant recently published literature (Rossman et al. 2007, Suetrong et al. 2015, Norphanphoun et al. 2016, Hongsanan et al. 2017, Senanayake et al. 2017, Voglmayr et al. 2017, Yang et al. 2018, Fan et al. 2018a, b, 2020) (Table 1). Subsequent alignments for each gene were generated using MAFFT v.7 (Katoh and Standley 2013) and manually improved where necessary using MEGA v. 6 (Tamura et al. 2013). Novel sequences generated in the current study were deposited in GenBank (Table 1, Suppl. materials 1–3: Tables S1–S3) and the aligned matrices used for phylogenetic analyses were submitted to TreeBASE (www.treebase.org; accession number: S24893).

**Table 2.** Genes used in this study with PCR primers, primer DNA sequence, optimal annealing temperature and corresponding references.

Locus	Definition	Primers	Primer DNA sequence (5'–3')	Optimal annealing temp (°C)	References of primers used
ITS	internal transcribed spacer of ribosomal RNA	ITS1	TCCGTAGGTGAACCTGCGG	51	White et al. 1990
		ITS4	TCCTCCGCTTTTGATATGC		
LSU	large subunit of ribosomal RNA	LR0R	ACCCGCTGAACTTAAGC	55	Vilgalys and Hester 1990
		LR7	TACTACCACCAAGATCT		
*cal*	Calmodulin	CAL-228F	GAGTTCAAGGAGGCCTTCTCCC	55	Carbone and Kohn 1999
		CAL-737R	CATCTTTCTGGCCATCATGG		
*rpb2*	RNA polymerase II second largest subunit	RPB2-5F	GA(T/C)GA(T/C)(A/C)G(A/T)GATCA(T/C)TT(T/C)GG	52	Liu et al. 1999
		RPB2-7cR	CCCAT(A/G)GCTTG(T/C)TT(A/G)CCCAT		
*his3*	histone H3	CYLH4F	AGGTCCACTGGGTGGCAAG	58	Crous et al. 2004
		H3-1b	GCGGGCGAGCTGGATGTCCTT		Glass and Donaldson 1995
*tef-1α*	translation elongation factor 1-alpha	EF1-668F	CGGTCACTTGATCTACAAGTGC	55	Alves et al. 2008
		EF1-1251R	CCTCGAACTCACCAGTACCG		
*tub2*	beta-tubulin	Bt2a	GGTAACCAAATCGGTGCTGCTTTG	55	Glass and Donaldson 1995
		Bt2b	ACCCTCAGTGTAGTGACCCTTGGC		

### Phylogenetic analyses

To infer the first phylogenetic relationships at the family level, an initial alignment combining the here generated and available ITS, LSU, *rpb2* and *tef1-α* sequences was compiled following Fan et al. (2018a). This alignment was analyzed based on Maximum Parsimony (MP), Maximum Likelihood (ML), and Bayesian Inference (BI) methods.

The MP analysis was conducted using a heuristic search (1,000 bootstrap) by PAUP v. 4.0b10 (Swofford 2003). The MP analysis was conducted with random sequence additions as option to stepwise-addition (1,000 bootstrap replicates and one tree held at each addition step), and maxtrees limited to 100 by replicate. The tree bisection and reconnection (TBR) was selected as option to the branch swapping algorithm (Swofford 2003). The branches of zero length were collapsed and all equally most parsimonious trees were saved. Other calculated parsimony scores were tree length (TL), consistency index (CI), retention index (RI) and rescaled consistency (RC). The ML analysis was performed using a GTR site substitution model, including a gamma-distributed rate heterogeneity and a proportion of invariant sites in PhyML v. 3.0 (Guindon et al. 2010). The BI analysis was conducted using the best-fit evolutionary models for each partitioned locus estimated in MrModeltest v. 2.3 (Posada and Crandall 1998) following the Akaike Information Criterion (AIC), with a Markov Chain Monte Carlo (MCMC) algorithm in MrBayes v. 3.1.2 (Ronquist and Huelsenbeck 2003). Two MCMC chains were run from random trees for 10 million generations and terminated when the average standard deviation of split frequencies dropped below 0.01. Trees were saved in each 1,000 generations. The first 25 % of trees were discarded at the burn-in phase of each analysis, and the Bayesian posterior probabilities (BPP) were calculated to assess the remaining trees (Rannala and Yang 1996). The MP bootstrap support (BS) equal to or above 50 were shown at the first and second position in branches. The branches with significant BPP equal to or above 0.95 were thickened in the phylogram.

In addition to the above analyses, we provided separate phylogenetic trees for two additional genera (*Dendrostoma* and *Diaporthe*) in Diaporthales, based on various gene regions (see below) including the same parameters as in the analyses described above. The branch support from MP and ML analyses was evaluated with a bootstrap support (BS) method of 1,000 replicates (Hillis and Bull 1993). Phylograms were plotted in Figtree v. 1.4.4 (http://tree.bio.ed.ac.uk/software/figtree) and edited in Adobe Illustrator CS6 v.16.0.0 (https://www.adobe.com/cn/products/illustrator.html).

## Results

### Phylogenetic analysis

The combined matrix (ITS, LSU, *rpb2* and *tef1-α*) of Diaporthales included 198 ingroup accessions (15 from the current study and 183 retrieved from GenBank) and two outgroup taxa. The aligned matrix comprised 4,047 characters including gaps (773 characters for ITS, 1,190 for LSU, 1,114 for *rpb2* and 970 for *tef1-α*), of which 2,002 characters were constant, 158 variable characters were parsimony-uninformative and 1,887 characters were variable and parsimony-informative. MP analyses generated 100 parsimonious trees of which the first tree is presented in Fig. 2 (TL = 12,631, CI = 0.313, RI = 0.792, RC = 0.248). The tree topologies of ML and BI analyses were mostly similar to the generated MP tree. The 15 isolates obtained in this study were clustered within the families Diaporthaceae, Erythrogloeaceae, Juglanconidaceae and Melanconidaceae in Diaporthales (Fig. 2). To delimitate to the species level, phylogenetic trees for *Dendrostoma* and *Diaporthe* were constructed separately based on different DNA datasets.

**Figure 2.**
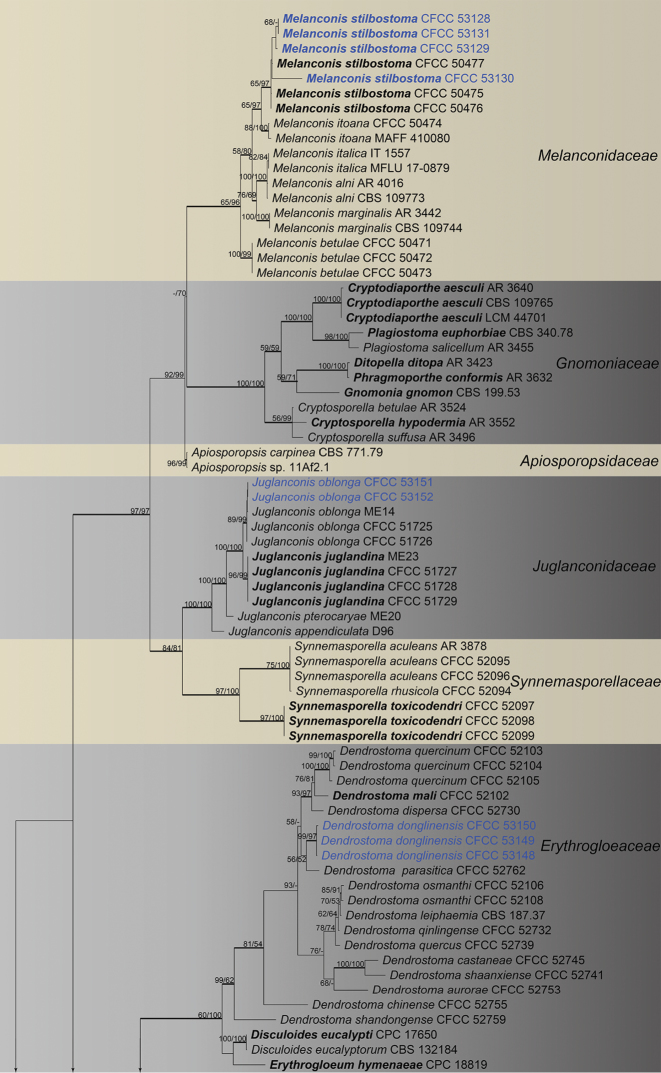
Phylogram of Diaporthales based on combined ITS, LSU, *rpb2* and *tef1-α* genes. The MP and ML bootstrap support values above 50 % are shown at the first and second position, respectively. Thickened branches represent posterior probabilities above 0.95 from the BI. Ex-type strains are in bold. Strains from the current study are in blue.

**Figure 2.**
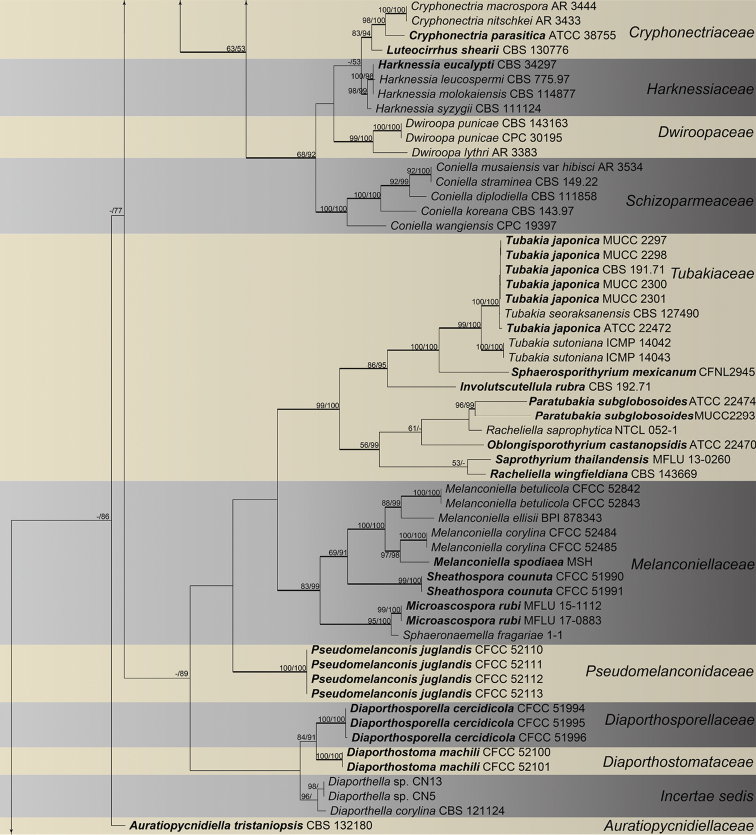
Continued.

**Figure 2.**
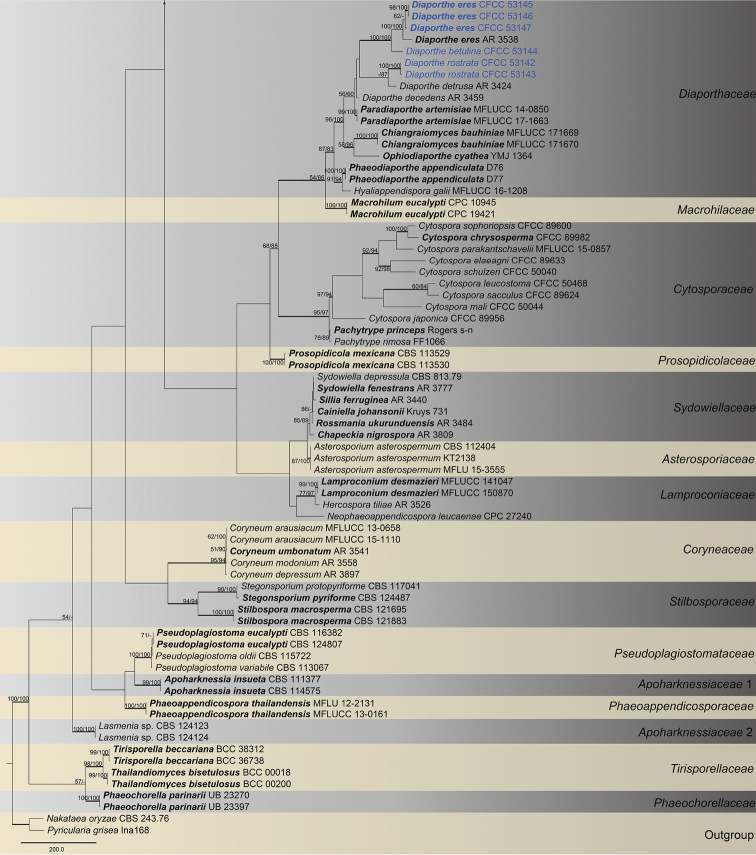
Continued.

For the genus *Diaporthe* (Diaporthaceae), a concatenated ITS, *cal*, *his3*, *tef1-α* and *tub2* matrix was produced with 201 ingroup accessions (6 from this study and 195 retrieved from GenBank). The combined matrix comprised 3,237 characters including gaps (544 characters for ITS, 593 for *cal*, 587 for *his3*, 645 for *tef1-α* and 868 for *tub2*) of which 1,330 characters were constant, 442 variable characters parsimony-uninformative and 1,465 characters variable and parsimony-informative. The MP analysis generated 100 parsimonious trees and the first tree is presented in Fig. 3 (TL = 12,978, CI = 0.280, RI = 0.712, RC = 0.199). The isolates of *Diaporthe* clustered in three different clades, corresponding to the three known species in this genus. The second combined matrix (*cal*, *tef1-α* and *tub2*) focusing on the *Diaporthe eres* complex included 56 ingroup accessions (4 from this study and 52 retrieved from GenBank). The concatenated matrix comprised 1,198 characters including gaps (405 for *cal*, 363 for *tef1-α* and 430 for *tub2*) of which 933 characters were constant, 112 variable characters parsimony-uninformative and 153 characters variable and parsimony-informative. The MP analysis generated 100 parsimonious trees of which the first is presented in Fig. 4 (TL = 415, CI = 0.701, RI = 0.882, RC = 0.618). The tree topologies of the ML and BI analyses were almost similar to the MP tree.

**Figure 3.**
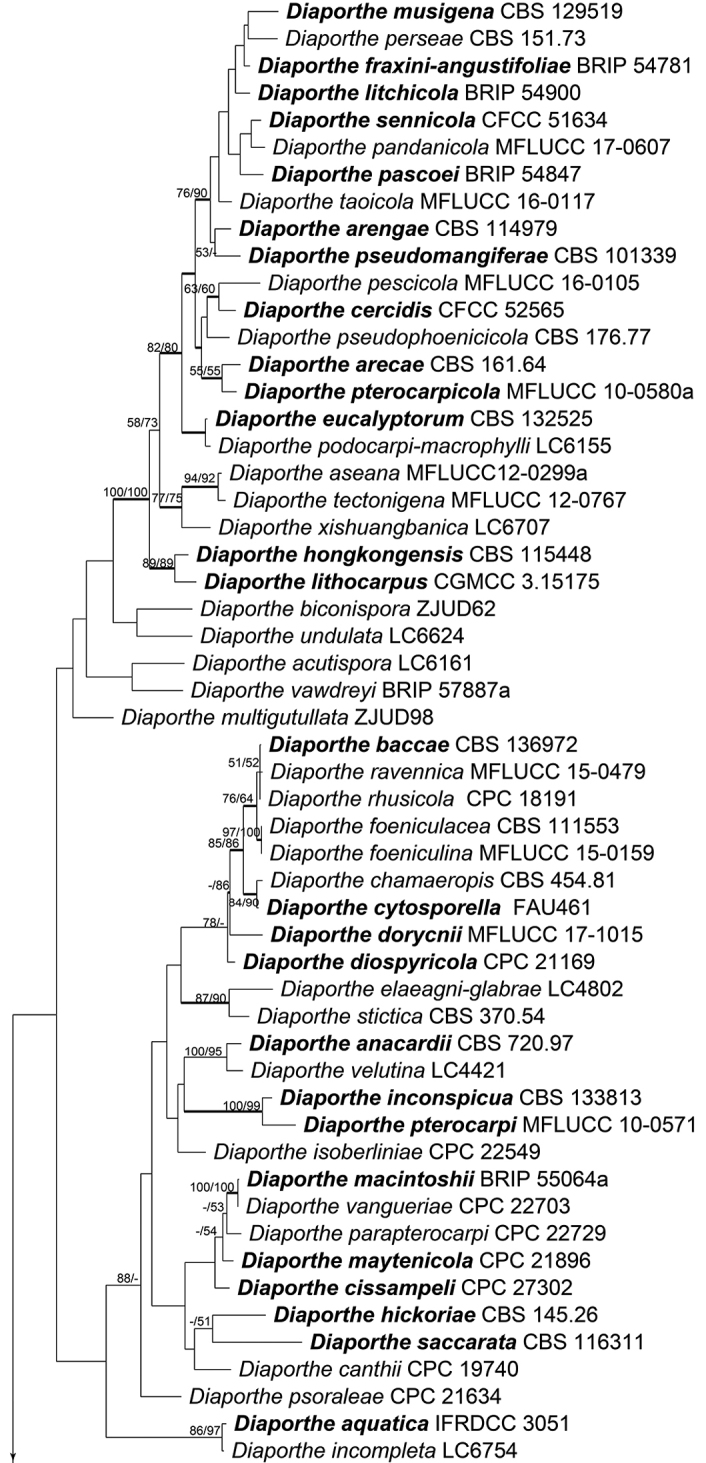
Phylogram of *Diaporthe* based on combined ITS, *tef1-α*, *tub2*, *cal* and *his3* genes. The MP and ML bootstrap support values above 50 % are shown at the first and second positions, respectively. Thickened branches represent posterior probabilities above 0.95 from the BI. Ex-type strains are in bold. Strains from the current study are in blue.

**Figure 3.**
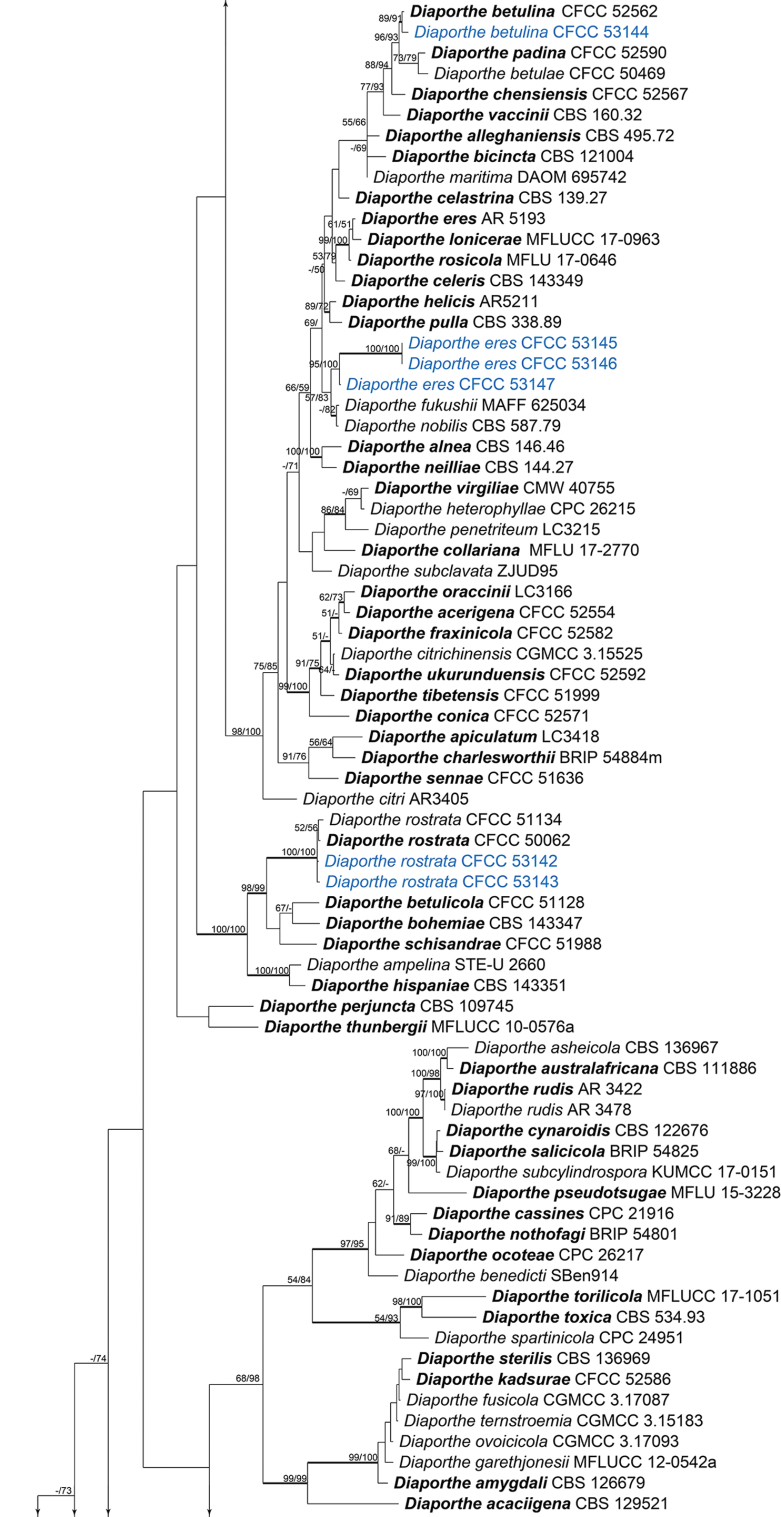
Continued.

**Figure 3.**
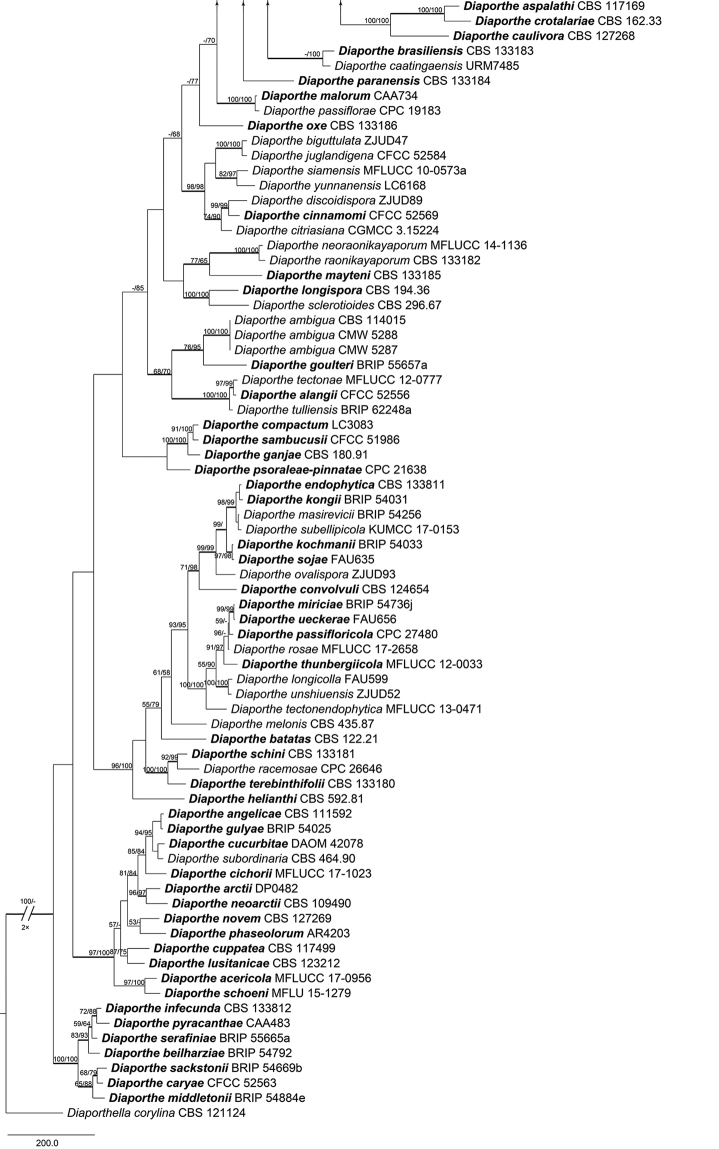
Continued.

**Figure 4.**
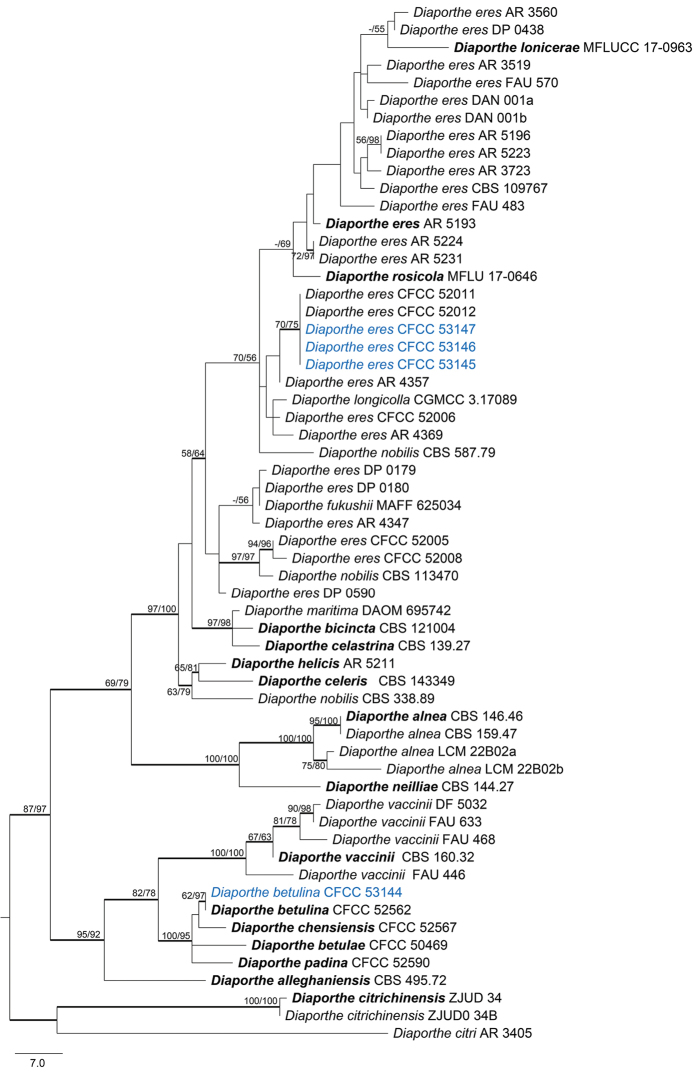
Phylogram of *Diaporthe eres* complex based on combined *cal*, *tef1-α* and *tub2* genes. The MP and ML bootstrap support values above 50 % are shown at the first and second positions, respectively. Thickened branches represent posterior probabilities above 0.95 from BI. Ex-type strains are in bold. Strains from the current study are in blue.

**Figure 5.**
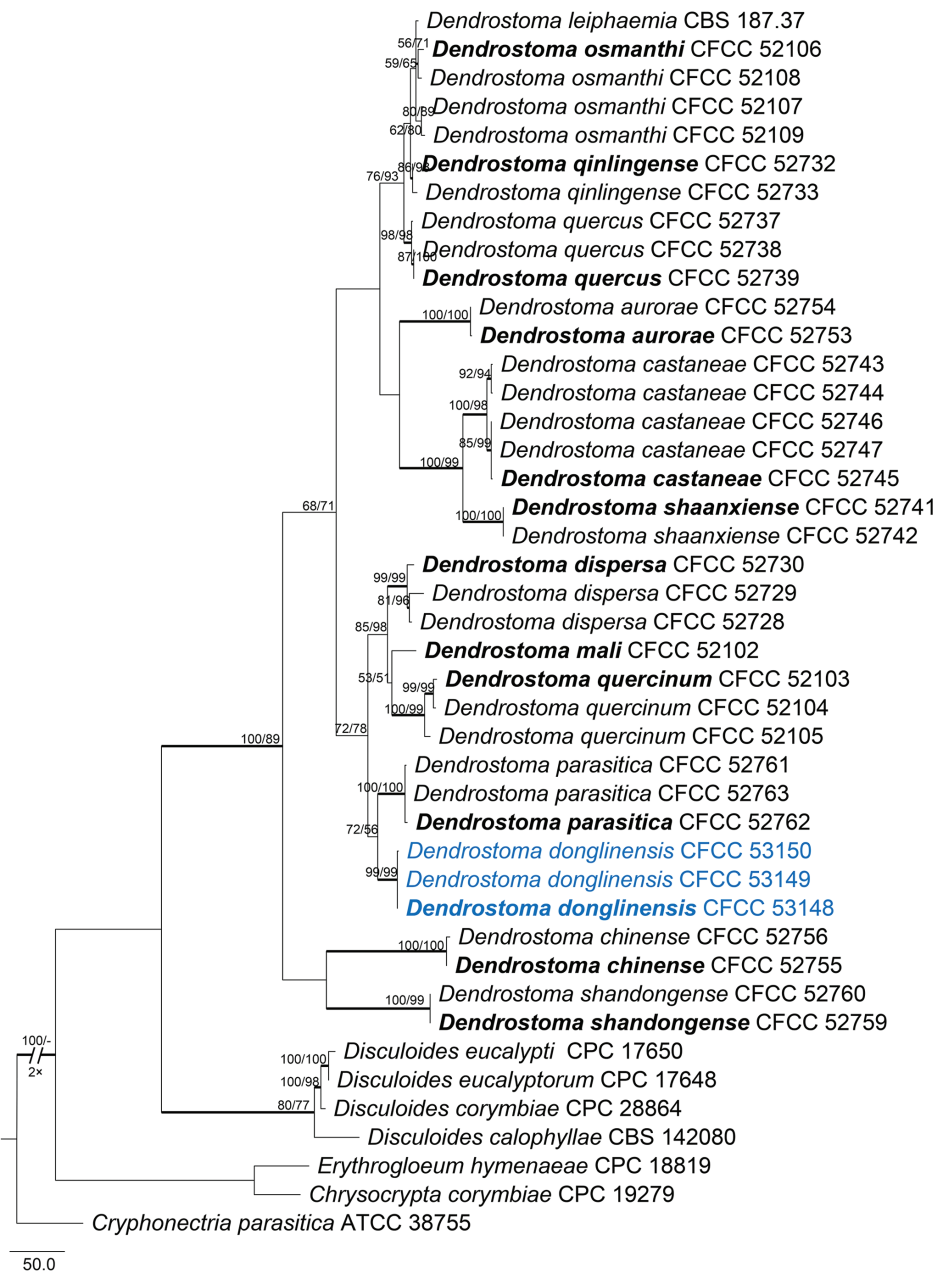
Phylogram of *Dendrostoma* based on combined ITS, *rpb2* and *tef1-α* genes. The MP and ML bootstrap support values above 50 % are shown at the first and second positions, respectively. Thickened branches represent posterior probabilities above 0.95 from the BI. Ex-type strains are in bold. Strains from the current study are in blue.

For the genus *Dendrostoma* (Erythrogloeaceae), ITS, *rpb2* and *tef1-α* alignments were concatenated, including 42 ingroup accessions (three from this study and 39 retrieved from GenBank) was produced. The full matrix comprised 2,400 characters including gaps (561 characters for ITS, 1,078 for *rpb2* and 761 for *tef1-α*), of which 1,486 characters are constant, 231 variable characters are parsimony-uninformative and 683 characters are variable and parsimony-informative. The only parsimonious tree generated in MP analyses is presented in Fig. 5 (TL = 1,691, CI = 0.707, RI = 0.835, RC = 0.591). Tree topologies of ML and BI analyses were mostly similar to the MP tree. Three isolates of *Dendrostoma* represented a monophyletic clade with high support value (MP/Ml/BI = 99/99/1) (marked in blue in Fig. 5).

### Taxonomy

#### Diaporthaceae

Taxon classificationFungiDiaporthalesDiaporthaceae

Höhn. ex Wehm., Am. J. Bot. 13: 638 (1926)

BDC2B761-41C9-5A30-8DC3-06C301546933

##### Type genus.

*Diaporthe* Nitschke, Pyrenomyc. Germ. 2: 240 (1870).

##### Notes.

Diaporthaceae was introduced by von Höhnel (1917) and subsequently involved in confusing the taxonomy due to many genera with wide variation of morphological characters and the majority without culture or DNA phylogeny. Senanayake et al. (2017, 2018) accepted 14 genera in Diaporthaceae, including *Allantoporthe*, *Apioporthella*, *Chaetoconis*, *Chiangraiomyces*, *Diaporthe*, *Hyaliappendispora*, *Leucodiaporthe*, *Mazzantia*, *Ophiodiaporthe*, *Paradiaporthe*, *Phaeocytostroma*, *Phaeodiaporthe*, *Pustulomyces*, and *Stenocarpella*.

#### Diaporthe

Taxon classificationFungiDiaporthalesDiaporthaceae

Nitschke, Pyrenomyc. Germ. 2: 240 (1870)

F6E6BBBF-00A1-59EC-8159-AE60379B2798

##### Type species.

*Diaporthe eres* Nitschke, Pyrenomyc. Germ. 2: 245 (1870).

##### Notes.

The genus *Diaporthe* (syn. *Phomopsis*) was established by Nitschke (1870). The identification of *Diaporthe* was confused due to the historical species recognition criteria based on overlapped morphology, culture characteristics and host affiliation (Dissanayake et al. 2017). The phylogenetic analysis recommended to delimitate taxa to the species level was first proposed by Udayanga et al. (2012) and later modified to include concatenated alignments of ITS, *cal1*, *his3*, *tef1-α*, *tub2* (Gomes et al. 2013). More than 1,050 epithets for *Diaporthe* and 950 for *Phomopsis* are listed in Index Fungorum (August 2019). Dissanayake et al. (2017) provided most type/ex-type species details and phylogenetic frame with 172 species in this genus. Yang et al. (2018) summarized 15 species of *Diaporthe* associated with dieback disease of tree hosts in China and introduced 12 new species.

#### Diaporthe betulina

Taxon classificationFungiDiaporthalesDiaporthaceae

C.M. Tian & Q. Yang, Mycokeys 39: 97 (2018)

32597FDF-3E3B-5A57-87A6-30A48E50FEDD

##### Description.

See Yang et al. (2018).

##### Material examined.

CHINA, Beijing City, Mentougou District, Mount Dongling, Xiaolongmen Forestry Centre (39°59'23.58"N, 115°27'05.00"E), from branches of *Betula dahurica* Pall., 17 Aug. 2017, H.Y. Zhu & X.L. Fan, deposited by X.L. Fan, CF 2019831, living culture CFCC 53144.

##### Notes.

Yang et al. (2018) described *Diaporthe betulina* from cankers of *Betula* spp. in Heilongjiang Province. The only strain CFCC 53144 representing *D. betulina* clusters in a well-supported clade and appear most closely related to *D. betulae*, which was also isolated from *Betula platyphylla* in Sichuan Province (Du et al. 2016). *Diaporthe betulina* (strain CFCC 52562) differs from *D. betulae* by its slender alpha conidia (2.5–3 vs. 3–4 μm) (Du et al. 2016), and 13 bp for ITS, 7 bp for cal, 19 bp for his, 12 bp for tef and 6 bp for tub2 based on alignment of the concatenated five-gene deposited in TreeBASE (S24893). Both morphology and sequence data confirmed that our isolates belong to this species.

#### Diaporthe eres

Taxon classificationFungiDiaporthalesDiaporthaceae

Nitschke, Pyrenomyc. Germ. 2: 245 (1870)

A72E8DA0-A4AD-5A4B-86FB-9D5A9FE36DAE

Fig. 6

##### Description.

Sexual morph: not observed. Asexual morph: Pycnidial stromata immersed in bark, scattered, slightly erumpent through the bark surface, unilocular, with a conspicuous central column. Central column beneath the disc more or less conical, pale grey with yellow. Ectostromatic disc orange, elliptical, 160–300 μm in diam., with one ostiole per disc. Ostiole dark brown to black, at the same level as or slightly above the disc surface, 70–80 μm in diam. Locule single, 210–260 μm in diam. Conidiophores cylindrical, hyaline, unbranched, straight or slightly curved, tapering towards the apex, 12–13.5 × 2–3 μm. Conidiogenous cells enteroblastic, phialidic. Alpha conidia hyaline, aseptate, smooth, ellipsoidal, biguttulate, rounded at both ends, 6.5–8.5 × 2.5–3 (av. = 7.3± 0.5 × 2.8 ± 0.3, n = 30) μm. Beta conidia were not observed.

##### Culture characteristics.

Cultures on PDA are initially white, growing up to 4 cm in diam. after 3 days, and becoming yellow green to brown after 7–10 days. Colonies are ﬂat felty with a thick texture at the marginal area, with a thin texture at the center, abundant aerial mycelium, sterile.

##### Material examined.

CHINA, Beijing City, Mentougou District, Mount Dongling, Xiaolongmen Forestry Centre (39°58'06.45"N, 115°26'48.36"E), from branches of *Prunus davidiana* (Carr.) Franch., 20 Aug. 2017, H.Y. Zhu & X.L. Fan, deposited by X.L. Fan, CF 2019808, living culture CFCC 53146; *ibid.* CF 2019858, living culture CFCC 53145. CHINA, Beijing City, Mentougou District, Mount Dongling, Xiaolongmen Forestry Centre (39°57'47.49"N, 115°29'20.52"E), from branches of *Juglans regia* L., 20 Aug. 2017, H.Y. Zhu & X.L. Fan, deposited by X.L. Fan, CF 2019801, living culture CFCC 53147.

##### Notes.

*Diaporthe eres* is the type species of *Diaporthe*, and is also the most common species causing canker disease on a wide range of hosts (Gomes et al. 2013, Udayanga et al. 2014, Dissanayake et al. 2017, Yang et al. 2018). Our isolates are associated with canker disease of *Prunus davidiana* in China, which belong to the *Diaporthe eres* species complex (Fig. 4). Fan et al. (2018c) treated many *Diaporthe* species as *D. eres*, and showed the combined *cal*, *tef1-α* and *tub2* genes provide a better topology than the combined five-gene phylogeny for the *D. eres* complex. Both sequence data and morphology confirm that our isolates belong to this species (Fig. 4).

**Figure 6.**
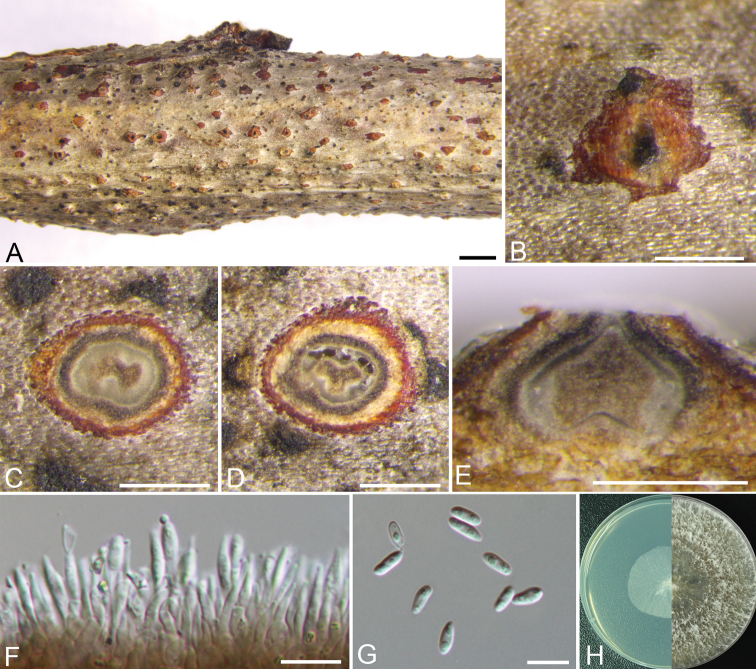
Morphology of *Diaporthe eres* from *Prunus davidiana* (CF 2019808). **A, B** Habit of conidiomata on twig **C, D** transverse section of conidioma **E** longitudinal section through conidioma **F** conidiophores and conidiogenous cells **G** alpha conidia **H** colonies on PDA at 3 days (left) and 30 days (right). Scale bars: 1mm (**A**); 250μm (**B–E**); 10 μm (**F, G**).

#### Diaporthe rostrata

Taxon classificationFungiDiaporthalesDiaporthaceae

C.M. Tian, X.L. Fan & K.D. Hyde, Mycological Progress 14: 82 (2015)

9B7256D6-25DF-555C-B596-AD03F9161B0F

≡ Diaporthe juglandicola C.M. Tian & Q. Yang. Mycosphere 8(5): 821 (2017)

##### Description.

See Fan et al. (2015).

##### Material examined.

CHINA, Beijing City, Mentougou District, Mount Dongling, Xiaolongmen Forestry Centre (39°57'54.68"N, 115°27'45.27"E), from branches of *Juglans mandshurica* Maxim., 22 Aug. 2017, H.Y. Zhu & X.L. Fan, deposited by X.L. Fan, CF 2019807, living culture CFCC 53142; *ibid.* CF 2019910, living culture CFCC 53143.

##### Notes.

Fan et al. (2015) introduced *Diaporthe rostrata* from *Juglans mandshurica* causing walnut dieback in China. Yang et al. (2017) introduced *D. juglandicola* as a sister clade with *D. rostrata*, but it has no conspicuous rostrate necks on the bark. However, we recommend to treat *D. juglandicola* as a synonym of *D. rostrate*, based on the same host species, and lacking of phylogenetic support to separate them after involving our current materials (CF 2019807 and CF 2019910) with conspicuous rostrate necks.

#### Erythrogloeaceae

Taxon classificationFungiDiaporthalesDiaporthaceae

Senan., Maharachch. & K.D. Hyde, Stud. Mycol. 86: 258 (2017)

0596547C-D614-5C1A-AE60-9902D8DAFF25

##### Type genus.

*Erythrogloeum* Petr. Sydowia 7: 378 (1953).

##### Notes.

The family *Erythrogloeaceae* was recently introduced by Senanayake et al. (2017) based on ITS, LSU, *rpb2* and *tef1-α*, and included four genera (*Chrysocrypta*, *Dendrostoma*, *Disculoides* and *Erythrogloeum*) (Fan et al. 2018a, Senanayake et al. 2018).

#### Dendrostoma

Taxon classificationFungiDiaporthalesDiaporthaceae

X.L. Fan & C.M. Tian, Persoonia 40: 124 (2018)

55280BE3-851F-5CC5-8B91-BC6EB182F7ED

##### Type species.

*Dendrostoma mali* X.L. Fan & C.M. Tian, Persoonia 40: 124 (2018).

##### Notes.

*Dendrostoma* was introduced by Fan et al. (2018a) as a phytopathogenic genus, causing canker diseases on several economic hardwoods such as *Malus spectabilis*, *Osmanthus fragrans* and *Quercus acutissima*. Jiang et al. (2019b) accepted 14 species of *Dendrostoma* using a concatenated matrix of four genes (ITS, LSU, *rpb2* and *tef1-α*), including 10 new species associated with chestnut and oak canker disease in China. Here we recommend a set of three genes (ITS, *rpb2* and *tef1-α*) to separate species of this genus.

#### Dendrostoma donglinensis

Taxon classificationFungiDiaporthalesDiaporthaceae

H.Y. Zhu & X.L. Fan sp. nov.

516759AC-FE6A-5D61-B47A-C24513F51EF9

832194

Fig. 7

##### Etymology.

Named after the location where it was collected, Mount Dongling.

##### Holotype.

CHINA, Beijing City, Mentougou District, Mount Dongling, Xiaolongmen Forestry Centre (39°58'19.62"N, 115°26'51.27"E), from branches of *Quercus mongolica* Fisch. ex Ledeb., 18 Aug. 2017, H.Y. Zhu & X.L. Fan, deposited by X.L. Fan, holotype CF 2019903, ex-type living culture CFCC 53148.

##### Description.

Sexual morph: not observed. Asexual morph: Pycnidial stromata immersed in the bark, scattered, erumpent through the surface of bark, unilocular, with a conspicuous central column. Central column beneath the disc more or less conical, yellow. Conceptacle absent. Ectostromatic disc hyaline, circular to ovoid, 750–1190 µm in diam., with a single ostiole per disc. Ostiole grey to black, at the same level as the disc surface, 240–270 μm in diam. Locule single, circular to irregular, undivided, 550–750 µm in diam. Conidiophores hyaline, unbranched, approximately cylindrical. Conidiogenous cells enteroblastic, phialidic. Conidia hyaline, fusoid, acute at each end, smooth or occasional not smooth, aseptate, 16.5–20.5 × 2–3.5 (av. = 18 ± 1.1 × 3 ± 0.3, n = 30) μm.

##### Culture characteristics.

Cultures on PDA are initially white, growing slowly to 2 cm in diam. after 3 days and 4 cm after 14 days, becoming salmon in the center after 7–10 days. Growth stops when colony reaches 8 cm and cultures becoming salmon to honey after the 30 days. Colonies are felty with a uniform texture; sterile.

##### Additional material examined.

CHINA, Beijing City, Mentougou District, Mount Dongling, Xiaolongmen Forestry Centre (39°58'19.62"N, 115°26'51.27"E), from branches of *Quercus mongolica* Fisch. ex Ledeb., 18 Aug. 2017, H.Y. Zhu & X.L. Fan, deposited by X.L. Fan, CF 2019887, living culture CFCC 53149; *ibid.* CF 2019805, living culture CFCC 53150.

##### Notes.

*Dendrostoma donglinensis* is associated with canker disease of *Quercus mongolica* in China. It can be distinguished from its closest relative *D. parasiticum* by its fusoid, acute at each end and larger conidia (16.5–20.5 × 2–3.5 vs. 9.3–11.7 ×2.8–3.3 μm). The isolates are phylogenetically distinct from all other available strains of *Dendrostoma* included in this study and we therefore describe this species as new, based on DNA sequence data and morphology.

**Figure 7.**
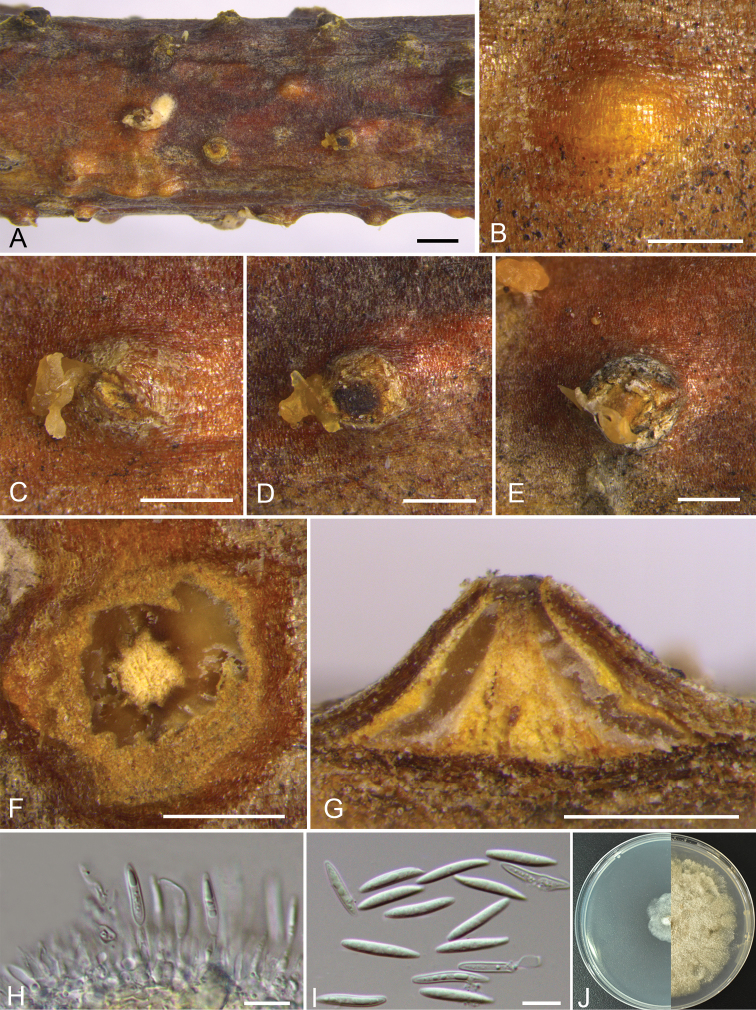
Morphology of *Dendrostoma donglinensis* from *Quercus mongolica* (CF 2019903). **A–E** Habit of conidiomata on twig **F** transverse section of conidioma **G** longitudinal section through conidioma **H** conidiophores and conidiogenous cells **I** conidia **J** colonies on PDA at 3 days (left) and 30 days (right). Scale bars: 1mm (**A**); 500 μm (**B–G**); 10 μm (**H, I**).

#### Juglanconidaceae

Taxon classificationFungiDiaporthalesDiaporthaceae

Voglmayr & Jaklitsch, Persoonia 38: 142 (2017)

E453A3C2-0D04-5FD6-980A-4B0429981B50

##### Type genus.

*Juglanconis* Voglmayr & Jaklitsch, Persoonia 38: 142 (2017).

##### Notes.

Juglanconidaceae was introduced by Voglmayr et al. (2017), including a single genus *Juglanconis*.

#### Juglanconis

Taxon classificationFungiDiaporthalesDiaporthaceae

Voglmayr & Jaklitsch, Persoonia 38: 142 (2017)

AEF8C38F-3E81-5CE8-99F0-A7AADD44F84D

##### Type species.

*Juglanconis juglandina* (Kunze) Voglmayr & Jaklitsch, *Persoonia* 38: 144 (2017).

##### Notes.

*Juglanconis* was introduced by Voglmayr et al. (2017) to accommodate previous *Melanconium juglandinum*, *M. oblongum* and *M. pterocaryae* based on morphology and DNA data of type materials. The genus is restricted to one host in Juglandaceae, which is identified by having perithecial ascomata, 8-spored asci with an apical ring, hyaline, bicelled ascospores in the sexual morph; and acervular conidiomata, brown conidia with gelatinous sheaths in asexual morph (Voglmayr et al. 2017). *Juglanconis* includes five species (*J. appendiculata*, *J. japonica*, *J. juglandina*, *J. oblonga* and *J. pterocaryae*) (Voglmayr et al. 2019), of which *J. juglandina* and *J. oblonga* are common pathogens in *Juglans* spp. in China (Fan et al. 2018b).

#### Juglanconis oblonga

Taxon classificationFungiDiaporthalesDiaporthaceae

(Berk.) Voglmayr & Jaklitsch Persoonia 38: 147 (2017)

3DB386CA-39C7-509D-8F8D-69A43E2BEDFC

≡ Melanconium oblongum Berk., Grevillea 2 (22): 153 (1874)
≡ Diaporthe juglandis Ellis & Everh., Proc. Acad. Nat. Sci. Philadelphia 45: 448 (1893)
≡ Melanconis juglandis (Ellis & Everh.) A.H. Graves, Phytopathology 13: 311 (1923)

##### Description.

See Fan et al. (2018b).

##### Material examined.

CHINA, Beijing City, Mentougou District, Mount Dongling, Xiaolongmen Forestry Centre (39°57'54.68"N, 115°27'45.27"E), from branches of *Juglans mandshurica* Maxim., 22 Aug. 2017, H.Y. Zhu & X.L. Fan, deposited by X.L. Fan, CF 2019906, living culture CFCC 53151; *ibid.* CF 2019909, living culture CFCC 53152.

##### Notes.

*Juglanconis oblonga* (previous *Melanconium oblongum*) is associated with canker disease of Juglandaceae hosts in North America and Southeast Asia (Graves 1923, Voglmayr et al. 2017, Fan et al. 2018b). This species is similar to *J. juglandina* in disease symptoms but can be distinguished by its longer conidia (22 × 12.5 compared to 20 × 13 μm) and DNA sequence data (Fan et al. 2018b). This species is a common pathogen causing walnut canker in China (Fan et al. 2018b).

#### Melanconidaceae

Taxon classificationFungiDiaporthalesDiaporthaceae

G. Winter, Rabenh. Krypt. -Fl., Edn 2 (Leipzig) 1(2): 764 (1886)

102DFBB1-A113-5982-8204-948A252E7B8B

##### Type genus.

*Melanconis* Tul. & C. Tul., Select. Fung. Carpol. (Paris) 2: 115 (1863).

##### Notes.

Melanconidaceae was introduced by Winter (1886) and has been subject to some confusion due to the overlap in morphological characters between genera and the absence of DNA sequence data supporting the family concept (Barr 1978). Castlebury et al. (2002) and Rossman et al. (2007) restricted this family to a single genus *Melanconis* based on LSU rDNA sequences, which was adapted by recent studies (Senanayake et al. 2017, Fan et al. 2018b).

#### Melanconis

Taxon classificationFungiDiaporthalesDiaporthaceae

Tul. & C. Tul., Select. Fung. Carpol. (Paris) 2: 115 (1863)

886BA9B8-18F1-5EFF-989A-440319D8BFFA

##### Type species.

*Melanconis stilbostoma* (Fr.) Tul. & C. Tul., Select. Fung. Carpol. (Paris) 2: 115 (1863).

##### Notes.

*Melanconis* was established by Tulasne & Tulasne (1863) based on *Sphaeria stilbostoma*. *Melanconis* has approximately 105 species epithets recorded in Index Fungorum (August 2019), but for most species no living cultures or DNA sequence data are available. Rossman et al. (2007) suggested that many of the species previously residing in *Melanconis* may belong elsewhere. *Melanconis* includes five species (*Melanconis alni*, *Ms. betulae*, *Ms. marginalis*, *Ms. itoana* and the type species *Ms. stilbostoma*), which were all restricted to the hosts in Betulaceae (Fan et al. 2016, 2018b).

#### Melanconis stilbostoma

Taxon classificationFungiDiaporthalesDiaporthaceae

(Fr.) Tul. & C. Tul., Select. Fung. Carpol. (Paris) 2: 115 (1863)

CEE1B45E-5AD9-5486-A4D9-2E19CB829AFC

##### Description.

See Fan et al. (2016).

##### Material examined.

CHINA, Beijing City, Mentougou District, Mount Dongling, Xiaolongmen Forestry Centre (39°59'23.58"N, 115°27'05.00"E), from branches of *Betula dahurica* Pall., 22 Aug. 2017, H.Y. Zhu & X.L. Fan, deposited by X.L. Fan, CF 2019832, living culture CFCC 53128; *ibid.* CF 2019833, living culture CFCC 53129. CHINA, Beijing City, Mentougou District, Mount Dongling, Xiaolongmen Forestry Centre (39°59'23.58"N, 115°27'05.00"E), from branches of *Betula* sp., 21 Aug. 2017, H.Y. Zhu & X.L. Fan, deposited by X.L. Fan, CF 2019871, living culture CFCC 53130; *ibid.* CF 2019911, living culture CFCC 53131.

##### Notes.

*Melanconis stilbostoma* is the type species of *Melanconis* and is thus far only known to occur on *Betula* spp. with a global distribution (Fan et al. 2016). *Betula dahurica*, *B. pendula*, *B. rotundifolia*, *B. tianschanica* and *B. platyphylla* are recorded as hosts for *Melanconis stilbostoma* in China (Zhuang 2005, Fan et al. 2016, 2018b).

## Discussion

In the present work six diaporthalean species were identified residing in four families (Diaporthaceae, Erythrogloeaceae, Juglanconidaceae and Melanconidaceae) in the order Diaporthales. These include five known species (*Diaporthe betulina*, *D. eres*, *D. rostrata*, *Juglanconis oblonga* and *Melanconis stilbostoma*), and one new species (*Dendrostoma donglinensis*). All specimens in the current study were collected from symptomatic branches and twigs associated with canker or dieback diseases. *Dendrostoma* (Erythrogloeaceae) species were isolated from *Quercus mongolica* (Fagaceae). *Juglanconis* (Juglanconidaceae) species were isolated from *Juglans mandshurica* (Juglandaceae) and *Melanconis* (Melanconidaceae) species were isolated from *Betula dahurica* (Betulaceae), which suggests these fungi are host specific. *Diaporthe* (Diaporthaceae) species were isolated from *Betula dahurica* (Betulaceae), *Juglans regia*, *J. mandshurica* (Juglandaceae), *Prunus davidiana* (Rosaceae) and *Quercus mongolica* (Fagaceae). This might indicate that *Diaporthe* species are less host specific.

The classification of Diaporthales presented here follows the previous studies (Castlebury et al. 2002, Rossman et al. 2007) and discoveries of new taxa from many other works (Suetrong et al. 2015, Dissanayake et al. 2017, Voglmayr et al. 2017, Senanayake et al. 2017, 2018). We performed frequently and used four genes (ITS, LSU, *rpb2* and *tef1-α*) to evaluate the 30 families in this order, but it was found to be confusing in some taxa such as *Apoharknessia* and *Lasmenia* in Apoharknessiaceae (Fig. 2). It suggests that more studies using a multiphasic approach are still needed to clarify some issues in this order. Diaporthales includes many phytopathogenic genera such as *Dendrostoma*, *Diaporthe*, *Melanconis* and *Juglanconis*, which have been reported causing canker disease of tree hosts in China (Fan et al. 2016, 2018b, Yang et al. 2018, Jiang et al. 2019b). The current study focuses on diaporthalean fungi in Mount Dongling of Beijing, which is considered as a biodiversity hotspot with a high diversity for fungal species and (Guo et al. 2008, Zhu et al. 2018). We hope that the descriptions and molecular data of diaporthalean fungi in this study could provide a resource for future studies in this region.

## Supplementary Material

XML Treatment for Diaporthaceae

XML Treatment for Diaporthe

XML Treatment for Diaporthe betulina

XML Treatment for Diaporthe eres

XML Treatment for Diaporthe rostrata

XML Treatment for Erythrogloeaceae

XML Treatment for Dendrostoma

XML Treatment for Dendrostoma donglinensis

XML Treatment for Juglanconidaceae

XML Treatment for Juglanconis

XML Treatment for Juglanconis oblonga

XML Treatment for Melanconidaceae

XML Treatment for Melanconis

XML Treatment for Melanconis stilbostoma

## Supplementary materials

Supplementary material 1 Table S1. Isolates and GenBank accession numbers used in the phylogenetic analyses of Diaporthales Data type: molecular data https://binary.pensoft.net/file/347626This dataset is made available under the Open Database License (http://opendatacommons.org/licenses/odbl/1.0/). The Open Database License (ODbL) is a license agreement intended to allow users to freely share, modify, and use this Dataset while maintaining this same freedom for others, provided that the original source and author(s) are credited.Haiyan Zhu, Meng Pan, Guido Bonthond, Chengming Tian, Xinlei Fan

Supplementary material 2 Table S2. Isolates and GenBank accession numbers used in the phylogenetic analyses of *Diaporthe* Data type: molecular data https://binary.pensoft.net/file/347627This dataset is made available under the Open Database License (http://opendatacommons.org/licenses/odbl/1.0/). The Open Database License (ODbL) is a license agreement intended to allow users to freely share, modify, and use this Dataset while maintaining this same freedom for others, provided that the original source and author(s) are credited.Haiyan Zhu, Meng Pan, Guido Bonthond, Chengming Tian, Xinlei Fan

Supplementary material 3 Table S3. Isolates and GenBank accession numbers used in the phylogenetic analyses of *Diaporthe eres* complex Data type: molecular data https://binary.pensoft.net/file/347628This dataset is made available under the Open Database License (http://opendatacommons.org/licenses/odbl/1.0/). The Open Database License (ODbL) is a license agreement intended to allow users to freely share, modify, and use this Dataset while maintaining this same freedom for others, provided that the original source and author(s) are credited.Haiyan Zhu, Meng Pan, Guido Bonthond, Chengming Tian, Xinlei Fan

Supplementary material 4 Table S4. Isolates and GenBank accession numbers used in the phylogenetic analyses of *Dendrostoma* Data type: molecular data https://binary.pensoft.net/file/347832This dataset is made available under the Open Database License (http://opendatacommons.org/licenses/odbl/1.0/). The Open Database License (ODbL) is a license agreement intended to allow users to freely share, modify, and use this Dataset while maintaining this same freedom for others, provided that the original source and author(s) are credited.Haiyan Zhu, Meng Pan, Guido Bonthond, Chengming Tian, Xinlei Fan
