# Supplementary material for: Diaporthalean fungi associated with canker and dieback of trees from Mount Dongling in Beijing, China
Source: MycoKeys. 2019 Oct 16;59:67–94. doi: 10.3897/mycokeys.59.38055 (PMC6811392; doi:10.3897/mycokeys.59.38055)
Supplement: Supplementary material 1 [file mycokeys-59-067-s004.docx]

| **Supplementary Table 4.** Isolates and GenBank accession numbers used in the phylogenetic analyses of *Diaporthe eres* complex. | | | | | | |
| --- | --- | --- | --- | --- | --- | --- |
| Species | Strain^1^ | Host | Origin | GenBank accession numbers | | |
|  |  |  |  | *cal* | *tef1-α* | *tub2* |
| *Diaporthe alleghaniensis* | CBS 495.72^T^ | *Betula alleghaniensis* | Canada | MH121426 | MH121544 | MH121584 |
| *Diaporthe alnea* | CBS 146.46^T^ | *Alnus* sp. | Netherlands | KC343250 | KC343734 | KC343976 |
|  | CBS 159.47 | *Alnus* sp. | NA | KC343251 | KC343735. | KC343977 |
|  | LCM 22B02a | *Alnus* sp. | USA | KJ435020 | KJ210557 | KJ420825 |
|  | LCM 22B02b | *Alnus* sp. | USA | KJ435021 | KJ210558 | KJ420826 |
| *Diaporthe betulae* | CFCC 50469 | *Betula platyphylla* | China | KT732997 | KT733016 | KT733020 |
| *Diaporthe betulina* | CFCC 52562^T^ | *Betula platyphylla* | China | MH121421 | MH121539 | MH121579 |
|  | **CFCC 53144** | *Betula dahurica* | Beijing, China | MN315462 | MN315474 | MN315470 |
| *Diaporthe bicincta* | CBS 121004^T^ | *Juglans* sp. | USA | KC343376 | KC343860 | KC344102 |
| *Diaporthe celastrina* | CBS 139.27^T^ | Celastrus scandens | NA | KC343289 | KC343773 | KC344015 |
| *Diaporthe celeris* | CBS 143349^T^ | *Vitis vinifera* | United Kingdom | MG281712 | MG281538 | MG281190 |
| *Diaporthe chensiensis* | CFCC 52567^T^ | *Abies chensiensis* | China | MH121426 | MH121544 | MH121584 |
| *Diaporthe citri* | AR 3405 | *Citrus* sp. | USA | KC843157 | KC843071 | KC843187 |
| *Diaporthe citrichinensis* | ZJUD 34^T^ | *Citrus unshiu* | China | NA | NA | KJ490396 |
|  | ZJUD0 34B | *Citrus* sp. | China | KJ435042 | KJ210562 | NA |
| *Diaporthe eres* | AR 3560 | *Viburnum lantana* | Austria | KJ435011 | JQ807351 | KJ420795 |
|  | DP 0438 | *Ulmus minor* | Austria | KJ435016 | KJ210553 | KJ420816 |
|  | AR 3519 | *Corylus avellana* | Austria | KJ435008 | KJ210547 | KJ420789 |
|  | FAU 570 | *Oxydendrum arboreum* | USA | KJ435025 | JQ807410 | KJ420794 |
|  | DAN 001a | *Daphne laureola* | France | KJ434994 | KJ210540 | KJ420781 |
|  | DAN 001b | *Daphne laureola* | France | KJ434995 | KJ210541 | KJ420782 |
|  | AR 5196 | *Ulmus* sp. | Austria | KJ435006 | KJ210554 | KJ420817 |
|  | AR 5223 | *Acer negundo* | Germany | KJ435000 | KJ210549 | KJ420830 |
|  | AR 3723 | *Rubus fruticosus* | Austria | KJ435024 | JQ807354 | KJ420793 |
|  | CBS 109767 | *Acer campestre* | Austria | KC343317 | KC343801 | KC344043 |
|  | FAU 483 | *Malus* sp. | Netherlands | KJ435022 | KJ210556 | KJ420827 |
|  | AR 5193^T^ | *Ulmus* sp. | Germany | KJ434999 | KJ210550 | KJ420799 |
|  | AR 5224 | *Hedera helix* | Germany | KJ435036 | KJ210551 | KJ420802 |
|  | AR 5231 | *Hedera helix* | Germany | KJ435038 | KJ210555 | KJ420818 |
|  | AR 4357 | *Zizyphus* sp. | South Korea | KJ435031 | JQ807360 | KJ420806 |
|  | AR 4369 | *Pyrus* sp. | South Korea | KJ435005 | JQ807366 | KJ420813 |
|  | DP 0179 | *Pyrus pyrifolia* | New Zealand | KJ435028 | JQ807383 | KJ420803 |
|  | DP 0180 | *Pyrus pyrifolia* | New Zealand | KJ435029 | JQ807384 | KJ420804 |
|  | AR 4347 | *Vitis sp.* | South Korea | KJ435030 | JQ807356 | KJ420805 |
|  | DP 0590 | *Pyrus pyrifolia* | New Zealand | KJ435037 | JQ807394 | KJ420810 |
|  | CFCC 52008 | *Juglans regia* | Beijing, China | MF279898 | MF279869 | MF279884 |
|  | CFCC 52005 | *Juglans regia* | Beijing, China | MF279899 | MF279870 | MF279885 |
|  | CFCC 52006 | *Juglans regia* | Beijing, China | MF279900 | MF279871 | MF279886 |
|  | CFCC 52011 | *Juglans regia* | Beijing, China | MF279894 | MF279865 | MF279880 |
|  | CFCC 52012 | *Juglans regia* | Beijing, China | MF279895 | MF279866 | MF279881 |
|  | **CFCC 53145** | *Prunus davidiana* | Beijing, China | NA | MN315476 | MN315472 |
|  | **CFCC 53146** | *Prunus davidiana* | Beijing, China | NA | MN315475 | MN315471 |
|  | **CFCC 53147** | *Juglans regia* | Beijing, China | NA | MN315477 | MN315473 |
| *Diaporthe fukushii* | MAFF 625034 | *Pyrus pyrifolia* | Japan | KJ435023 | NA | KJ420819 |
| *Diaporthe helici* | AR 5211^T^ | *Hedera helix* | France | KJ435043 | KJ210559 | KJ420828 |
| *Diaporthe longicolla* | CGMCC 3.17089 | *Lithocarpus glabra* | China | NA | KF576242 | KF576291 |
| *Diaporthe lonicerae* | MFLUCC 17-0963^T^ | *Lonicera* sp. | Italy | KY964116 | KY964146 | KY964073 |
| *Diaporthe maritima* | DAOM 695742^T^ | *Picea rubens* | Canada | NA | KU552022 | KU574616 |
| *Diaporthe neilliae* | CBS 144.27^T^ | *Spiraea* sp. | USA | KC343386 | KC343870 | KC344112 |
| *Diaporthe nobilis* | CBS 587.79 | *Pinus parviflora* var. *pentaphylla* | Japan | KC343395 | KC343879 | KC344121 |
|  | CBS 113470 | *Castanea sativa* | South Korea | KC343388 | KC343872 | KC344114 |
|  | CBS 338.89 | *Hedera helix* | Yugoslavia | KC343394 | KC343878 | KC344120 |
| *Diaporthe padina* | CFCC 52590^T^ | *Padus racemosa* | China | MH121443 | MH121567 | MH121604 |
| *Diaporthe rosicola* | MFLU 17-0646T | *Rosa* sp. | United Kingdom | MG829274 | MG829270 | MG843877 |
| *Diaporthe vaccinii* | DF 5032 | *Vaccinium corymbosum* | USA | KC849456 | JQ807380 | KC843225 |
|  | FAU 633 | *Vaccinium* sp. | USA | NA | JQ807413 | NA |
|  | FAU 468 | *Vaccinium* sp. | USA | KC849458 | NA | KC843227 |
|  | CBS 160.32T | *Oxycoccus macrocarpos* | USA | MH121426 | MH121544 | MH121584 |
|  | FAU 446 | *Vaccinium macrocarpon* | USA | KJ420882 | JQ807398 | NA |

Notes: CBS: Westerdijk Fungal Biodiversity Institute (CBS-KNAW Fungal Biodiversity Centre), Utrecht, The Netherlands; CFCC: China Forestry Culture Collection Centre, Beijing, China; CGMCC: China general Microbiological Culture Collection Centre; MFLU: Mae Fah Luang University herbarium, Thailand; MFLUCC: Mae Fah Luang University Culture Collection, Thailand; NA: not applicable. All the new isolates used in this study are indicated in bold type and the strains from generic type species are marked by an superscript (T).
